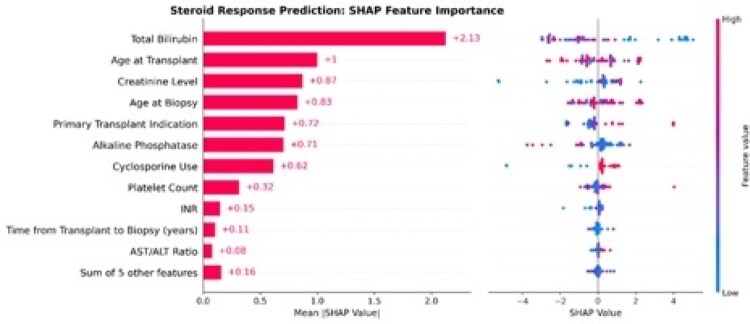# Poster Session I - A181 DEVELOPMENT OF AN IMPLEMENTABLE MULTIMODAL ARTIFICIAL INTELLIGENCE (AI) TOOL TO PREDICT HISTOLOGICAL RESPONSE IN LIVER TRANSPLANT RECIPIENTS WITH REJECTION

**DOI:** 10.1093/jcag/gwaf042.181

**Published:** 2026-02-13

**Authors:** A Chinnadurai, M Enrizky Brillian, G Azarfar, E Jaeckel, D Dodington, G Cazzaniga, A Gerussi, C McIntosh, M Bhat

**Affiliations:** University Health Network, Toronto, ON, Canada; University Health Network, Toronto, ON, Canada; University Health Network, Toronto, ON, Canada; University Health Network, Toronto, ON, Canada; University Health Network, Toronto, ON, Canada; Milano-Bicocca’s School of Medicine and Surgery, Monza, Italy; Milano-Bicocca’s School of Medicine and Surgery, Monza, Italy; University Health Network, Toronto, ON, Canada; University Health Network, Toronto, ON, Canada

## Abstract

**Background:**

T-cell mediated rejection (TCMR) occurs in approximately 30% of liver transplant recipients (LTR). While most respond to high dose steroids, 30% develop steroid-resistant rejection with poor graft outcomes. Early identification of non-responders would help in optimizing treatment of TCMR and improve graft outcomes.

**Aims:**

To develop a multimodal artificial intelligence (AI) model that integrates clinical and histological data to predict steroid responsiveness in TCMR

**Methods:**

We conducted a retrospective study of 55 adult LTRs who underwent liver biopsy-confirmed moderate-to-severe TCMR (Rejection Activity Index [RAI] ≥4) at a tertiary transplant center. We extracted liver pathology whole slide images (WSIs) with a typical size of 50,000 pixels into approximately 55,000 image patches and filtered patches using segmentation transformer (HOTSPoT) to retain only those containing portal tracts, reducing to 4,000 patches. Each patch was encoded as histological features using a Vision Transformer (UNI), resulting in 1,536-dimensional feature vectors. Clinical and laboratory data at time of rejection also collected.

We developed machine learning (ML) models to predict steroid responsiveness using both single- and multimodality feature sets. Single-modality used either histopathology-derived features or clinical variables, while multimodality models integrated both. Two fusion strategies were used: early fusion, combining pathology and clinical features prior to model training, and late fusion, integrating predictions from independent single-modality models. We also developed a 3-clicks user interface application, allowing users to input clinical data and pathology slides and the application automatically performs ROI classification and fusion prediction.

**Results:**

Clinical-only models showed limited predictive power (AUROC 0.52), whereas pathology-only models performed better (AUROC 0.61). Late fusion achieved the highest accuracy (AUROC 0.63). ROI classification yielded strong performance (AUROC 0.95) for predicting steroid responsiveness. Feature importance analysis highlighted histological features as primary drivers, with bilirubin, age at transplant, and creatinine as key clinical predictors.

**Conclusions:**

Our multimodal AI model integrates clinical and histopathological data to accurately predict steroid response in LTR facing TCMR. These tools have the potential to support early treatment decisions and thereby improve graft survival.

A181 Table 1: Model Performance on test set

Feature importance plot for clinical data classification using SHAP values.

Feature contributions were calculated using SHapley Additive exPlanations (SHAP). Higher average SHAP values indicate greater influence on the model’s prediction of steroid response. The sign of the SHAP value reflects the direction of association: for example, a SHAP value of –2.13 for total bilirubin suggests a negative relationship with steroid responsiveness, indicating that patients with elevated total bilirubin are less likely to respond to treatment.

**Funding Agencies:**

None